# Alkaline Mineral Complex Water Attenuates Transportation-Induced Hepatic Lipid Metabolism Dysregulation by AMPKα-SREBP-1c/PPARα Pathways

**DOI:** 10.3390/ijms252111373

**Published:** 2024-10-23

**Authors:** Linli Gan, Hongrui Guo, Qiyuan Yang, Xueke Zhou, Yue Xie, Xiaoping Ma, Liping Gou, Jing Fang, Zhicai Zuo

**Affiliations:** 1 College of Veterinary Medicine, Sichuan Agricultural University, Wenjiang, Chengdu 611130, China; ganlinli1995@163.com (L.G.); guohongrui@sicau.edu.cn (H.G.); stella0417@126.com (X.Z.); zhandegaokandey123@163.com (Y.X.); mxp886@sicau.edu.cn (X.M.); glping0827@163.com (L.G.); fangjing4109@163.com (J.F.); 2 Key Laboratory of Animal Diseases and Environmental Hazards of Sichuan Province, Sichuan Agriculture University, Wenjiang, Chengdu 611130, China; 3 Sichuan Academy of Grassland Sciences, Chengdu 611731, China; yqy979531@163.com

**Keywords:** alkaline mineral complex water, transportation, lipid metabolism, liver, AMPKα-SREBP-1c/PPARα pathway

## Abstract

Transportation, an unavoidable process in livestock farming, causes metabolic disorders in the body, which then lead to endocrine disruption, being immunocompromised, and growth suppression. Lipid metabolism dysregulation is a critical phenotype induced by transportation. The liver is a vital organ in lipid metabolism, with a role in both lipid synthesis and lipolysis. However, the specific mechanisms by which transportation affects hepatic lipid metabolism remain unclear. This study employed rats as a model to investigate the effects of transportation on hepatic lipid metabolism. Rats subjected to transportation showed altered serum lipid profiles, including decreased serum triglyceride (TG), low-density lipoprotein cholesterol (VLDL-C), and non-esterified fatty acid (NEFA) immediately after transportation (IAT) and serum total cholesterol (TC) on day 3, and increasing serum TG, TC, and low-density lipoprotein cholesterol (LDL-C) on day 10. Meanwhile, fatty droplets in the liver were also reduced at IAT and increased on days 3 and 10. Notably, transportation also affected hepatic-lipid-metabolism-related enzyme activities and signaling pathways, such as increased AMP-activated protein kinase alpha (AMPKα) phosphorylation and modulations in key proteins and genes related to lipid metabolism, decreased hepatic acetyl-CoA carboxylase (ACC) and fatty acid synthase (FAS) activities at IAT, and increased carnitine palmitoyl transferase 1 alpha (CPT-1α) at IAT and ACC and CPT-1α activities on days 3 and 10. Supplementation with alkaline mineral complex water (AMC) before and after transportation mitigated the adverse effects on hepatic lipid metabolism by modulating the AMPKα-SREBP-1c/PPARα pathway, enhancing lipid synthesis, and reducing the oxidative catabolism of fatty acids. AMC inhibited the transportation-induced activation of AMPKα and restored the balance of lipid-metabolism-related enzymes and pathways. These findings highlight AMC’s potential as a therapeutic intervention to alleviate transportation-induced lipid metabolism disorders, offering significant implications for improving animal welfare and reducing economic losses in livestock farming.

## 1. Introduction

Transportation, an essential procedure in livestock and poultry farming, stresses animals and results in economic losses [1,2]. These losses are estimated to represent approximately 5% to 10% of the total market value in certain livestock sectors [3]. During transportation, animals experience food and water deprivation, which affects energy metabolism [4,5], as evidenced by elevated blood glucose [6,7,8] and dyslipidemia [7,9,10,11]. Dyslipidemia in transported animals is characterized by alterations in lipid profiles, including decreased serum concentrations of triglyceride (TG), total cholesterol (TC), and low-density lipoprotein cholesterol (LDL-C) [5,9], or increased high-density lipoprotein cholesterol (HDL-C) and non-esterified fatty acid (NEFA) [7,10,12]. Despite these observations, the underlying mechanisms of these changes remain unclear. The liver plays a crucial role in lipid metabolism, serving as the central hub for fatty acid synthesis and the generation of lipoproteins to facilitate lipid circulation [13]. Regulating the expression of hepatic-lipid-metabolism-related genes and enhancing hepatic lipid metabolism help alleviate heat-stress-induced lipid metabolism disorders and reduce serum TG, TC, and LDL-C concentrations [14,15]. Thus, improving hepatic lipid metabolism regulation is significant in alleviating lipid metabolism disorders. However, direct evidence is still lacking in determining whether transportation affects hepatic lipid metabolism, and the precise mechanisms involved remain to be elucidated.

Alkaline mineral complex water (AMC) is an alkaline ionic solution comprising various metallic elements. Supplementing drinking water with AMC 3 days before and 30 days after transportation could activate lipid metabolism in transported beef cattle, as evidenced by increasing serum very low-density lipoprotein cholesterol (VLDL-C) levels and decreasing activities of fatty acid synthase (FAS) and acetyl-CoA carboxylase (ACC) [16], but the mechanism of AMC that activates lipid metabolism remains unclear.

The AMPK-SREBP-1c/PPARα pathway plays a crucial role in regulating lipid metabolism [17,18,19,20]. AMP-activated protein kinase alpha (AMPKα) has two catalytic subunit isoforms of AMPKα1 and AMPKα2, and their phosphorylation at the Thr172 position is critical for AMPK activation [19]. AMPKα1 is more ubiquitously expressed across various tissues, while AMPKα2 is predominantly found in skeletal muscle, the heart, and the liver, playing a key role in regulating energy metabolism in these tissues [21]. Peroxisome proliferator-activated receptor alpha (PPARα) and sterol regulatory element-binding protein-1c (SREBP-1c) are important regulators of fatty acid oxidation and de novo fatty acid synthesis in the liver [22,23]. SREBP-1c regulates the expression of key enzymes such as ACC and FAS [17,24,25], while PPARα controls the expression of genes involved in lipolysis, transport, and fatty acid oxidation, including carnitine palmitoyl transferase 1 (CPT-1) [26,27]. AMPKα activation inhibits lipogenesis through the suppression of SREBP-1c and upregulation of PPARα transcript levels [28,29], thereby reducing intracellular fat accumulation [30] and promoting fatty acid oxidation [31,32]. Therefore, the AMPKα-SREBP-1c/PPARα pathway may be a key point in studying lipid metabolism disorders caused by transportation.

Due to their metabolic similarities with larger mammals, ease of controlling experimental conditions, shorter cycles, and lower costs, rats are often regarded as ideal models for studying stress-induced metabolic disorders [33,34,35]. This study investigated the impact of transportation on hepatic lipid metabolism by examining changes in blood lipids, enzyme activities, and the AMPKα-SREBP-1c/PPARα pathway in rats as a research model. The aim was to elucidate the mechanisms through which transportation affects hepatic lipid metabolism and to explore the regulatory role of AMC. The findings are intended to provide a foundation for further research aimed at mitigating transportation-induced lipid metabolism disorders in livestock and poultry.

## 2. Results

### 2.1. Effect of AMC on Blood Lipid Metabolism in Transported Rats

**Figure 1:**
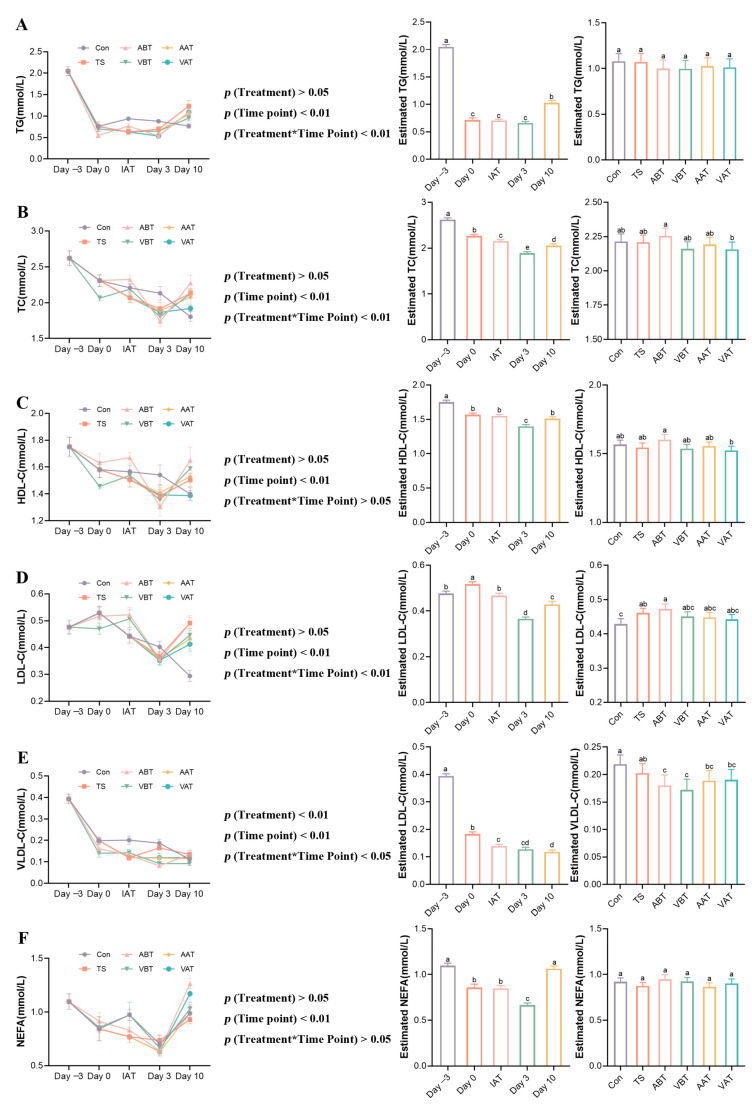
AMC alleviates transportation-induced lipid level disorders. (**A**–**F**) Curves of changes in blood lipid levels at different time points in rats with different treatments, estimates of blood lipid levels in rats with different treatments, and estimates of blood lipid levels in rats with different time points. (**A**) triglyceride (TG), (**B**) total cholesterol (TC), (**C**) high-density lipoprotein cholesterol (HDL-C), (**D**) low-density lipoprotein cholesterol (LDL-C), (**E**) very low-density lipoprotein cholesterol (VLDL-C), and (**F**) non-esterified fatty acid (NEFA). Rats were assigned to one of six groups: Con, TS, ABT, VBT, AAT, and VAT groups. Data are expressed as the mean ± standard error of the mean, where NEFA is *n* = 6 and the rest are *n* = 8. Different letters indicate significant differences (*p* < 0.05), and the same letters indicate non-significant differences (*p* > 0.05).

We determined the serum lipid metabolites to assess AMC’s effects on transported rats. As shown in Figure 1, the results of two-way ANOVA showed a significant effect of grouping on serum VLDL levels and a significant effect of time on lipid levels. And there was a significant interaction effect between time and grouping. In the Con group, serum TG decreased on day 0, slightly increased at IAT, and then gradually decreased on days 3 and 10. TC, HDL-C, and VLDL-C declined steadily, while LDL-C increased on day 0 and then gradually decreased. NEFA decreased on day 0, slightly increased at IAT, dropped on day 3, and rose on day 10. In the ABT group, TG, TC, and HDL-C decreased on day 0, slightly increased at IAT, dropped on day 3, and rose on day 10, while LDL-C increased on day 0 and decreased on days 3 and 10. VLDL-C and NEFA decreased on days 0 and 3, and then increased on day 10. In the VBT group, TG decreased on days 0 and 3, and then increased on day 10, while other lipid levels decreased on day 0, slightly increased at IAT, dropped on day 3, and rose on day 10. The TS group showed decreases in TG, TC, HDL-C, and LDL-C after transport, followed by gradual increases during recovery. VLDL-C and NEFA decreased at IAT, increased on day 3, and decreased on day 10. In the AAT group, TG increased during recovery, while other lipid levels first decreased on day 3 and then increased on day 10. The VAT group showed similar trends, with TG, TC, LDL-C, and NEFA decreasing on day 3 and rising on day 10, while HDL-C gradually decreased during recovery.

As shown in Figure S1, on day 0 (Figure S1A), TG, TC, VLDL-C, HDL-C, and NEFA significantly decreased in the Con group compared to day −3 (*p* < 0.05 or *p* < 0.01). Figure S1B shows that TC and VLDL-C in the VBT group were lower than in the Con group (*p* < 0.05). TC and HDL-C were higher in the ABT group than in the VBT group (*p* < 0.05). At IAT (Figure S1C), TG and VLDL-C levels significantly decreased in the TS group compared to the Con group (*p* < 0.05). Compared to the TS group, TC and LDL-C increased in the ABT group (*p* < 0.05). On day 3 (Figure S1D), lipid levels were similar between the Con and TS groups (*p* > 0.05). Compared to the TS group, VLDL-C levels were significantly lower in the ABT, VBT, and AAT groups (*p* < 0.05). On day 10 (Figure S1E), TG, TC, and LDL-C levels significantly increased in the TS group compared to the Con group (*p* < 0.01). Compared to the TS group, NEFA levels were higher in the ABT and VAT groups (*p* < 0.05), while TG in the VBT group and LDL-C in the VAT group were lower (*p* < 0.05). NEFA was significantly lower in the VBT group compared to the ABT group (*p* < 0.05). Pre- and post-transportation comparisons showed lower NEFA in the AAT group than in the ABT group (*p* < 0.05), and higher HDL-C in the VBT group compared to the VAT group (*p* < 0.05).

### 2.2. Effect of AMC on Hepatic Lipid Accumulation in Transported Rats

Oil-red O staining was used on frozen liver sections to assess AMC’s effect on lipid accumulation in transported rats. As shown in Figure S2A, on day 0, compared to day −3, staining revealed fewer fatty droplets (red) in the Con group (*p* < 0.01). The ABT group had more fatty droplets than the Con group, but the overall levels were similar to the Con group (*p* > 0.05).

As shown in Figure S2B, at IAT, the TS group had fewer fatty droplets compared to the Con group (*p* > 0.05). Fatty droplets increased in the ABT and VBT groups compared to the TS group (*p* < 0.01), but were similar between the ABT and VBT groups (*p* > 0.05).

As shown in Figure S2C, on day 3, the TS group had more fatty droplets than the Con group (*p* < 0.01). The ABT, VBT, AAT, and VAT groups had fewer droplets than the TS group. There were fewer fatty droplets in the ABT group than in the VBT group, and fewer in the AAT group than in the VAT group (*p* < 0.01). The ABT group had more droplets than the AAT group (*p* < 0.01).

As shown in Figure S2D, on day 10, the TS group had more fatty droplets than the Con group. The ABT, VBT, AAT, and VAT groups had fewer droplets than the TS group. The ABT group had fewer droplets than the VBT group (*p* < 0.01), and the AAT group had fewer than the VAT group. The ABT group had fewer droplets than the AAT group (*p* < 0.01).

### 2.3. Effects of AMC on Lipid-Metabolizing Enzymes in Transported Rats

**Figure 2:**
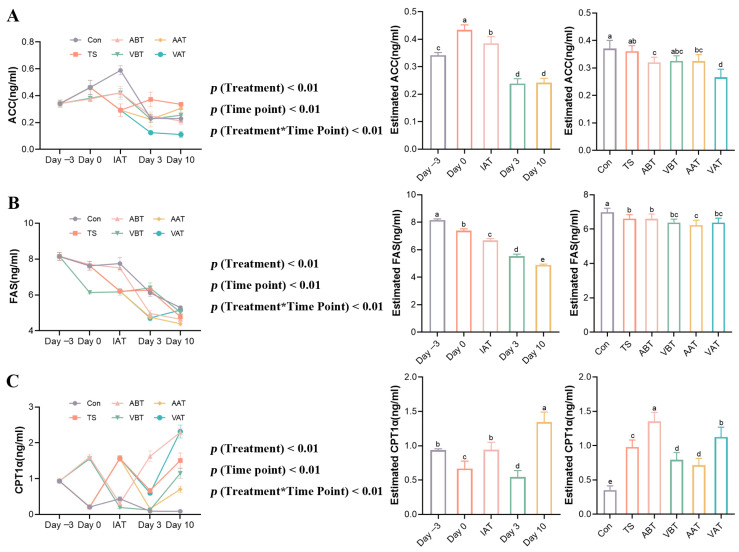
Effect of AMC on lipid-metabolizing enzymes in transported rats. (**A**–**C**) Curves of changes in lipid-metabolizing enzyme activity at different time points in rats with different treatments, estimates of lipid-metabolizing enzyme activity in rats with different treatments, and estimates of lipid-metabolizing enzyme activity in rats with different time points. (**A**) acetyl-CoA carboxylase (ACC), (**B**) fatty acid synthase (FAS), and (**C**) Carnitine palmitoyl transferase 1 alpha (CPT-1α). Rats were assigned to one of six groups: Con, TS, ABT, VBT, AAT, and VAT groups. Data are expressed as the mean ± standard error of the mean (*n* = 6). Different letters indicate significant differences (*p* < 0.05), and the same letters indicate non-significant differences (*p* > 0.05).

Hepatic-lipid-metabolism-related enzyme activities were measured to assess the effects of AMC in transported rats. As depicted in Figure 2, the results of two-way ANOVA showed that grouping and time had a significant effect on hepatic-lipid-metabolizing enzyme activities, and there was a significant interaction effect between time and grouping. In the Con group, ACC activity increased on day 0, peaked at IAT, and then gradually decreased on days 3 and 10. FAS and CPT-1α activities decreased on day 0, slightly increased at IAT, and then gradually declined on days 3 and 10. In the ABT and VBT groups, ACC activity followed the same pattern, peaking at IAT, while CPT-1α increased on day 0, dropped after transport, and then rose again, with the lowest point at IAT for ABT and on day 3 for VBT. FAS activity in the ABT group declined continuously, while in the VBT group, it decreased on day 0, rose at IAT and on day 3, and fell again on day 10. In the TS group, ACC and FAS decreased at IAT, rose on day 3, and declined on day 10, while CPT-1α increased at IAT, dropped on day 3, and rose again on day 10, peaking at IAT. In the AAT group, ACC and CPT-1α decreased on day 3 and rose on day 10, while FAS decreased on both days 3 and 10. In the VAT group, ACC decreased on days 3 and 10, while FAS and CPT-1α decreased on day 3 and rose on day 10.

As shown in Figure S3, on day 0 (Figure S3A), compared to day −3, ACC activity increased in the Con group (*p* < 0.05), while CPT-1α activities decreased (*p* < 0.01). As shown in Figure S3B, CPT-1α activity was significantly higher in the ABT and VBT groups than in the Con group (*p* < 0.01), while FAS activity decreased significantly in the VBT group (*p* < 0.01). FAS activity in the ABT group was higher than in the VBT group (*p* < 0.01). At IAT (Figure S3C), hepatic ACC and FAS activities were significantly reduced in the TS group compared to the Con group, while CPT-1α activity increased (*p* < 0.01). In the ABT and VBT groups, ACC and FAS activities (in ABT) were significantly higher than in the TS group, while CPT-1α activity decreased (*p* < 0.05 or *p* < 0.01). FAS activity in the ABT group was higher than in the VBT group (*p* < 0.01). On day 3 (Figure S3D), ACC and CPT-1α activities were significantly higher in the TS group compared to the Con group (*p* < 0.01). Compared to the TS group, ACC activity in the ABT, VBT, AAT, and VAT groups; FAS in the ABT, AAT, and VAT groups; and CPT-1α in the VBT and AAT groups were significantly decreased (*p* < 0.05), while CPT-1α in the ABT group increased (*p* < 0.01). FAS activity in the ABT group was lower and CPT-1α was higher than in the VBT group (*p* < 0.01). In the AAT group, ACC activity was higher and CPT-1α was lower than in the VAT group (*p* < 0.01). CPT-1α activity was significantly higher in the ABT group than in the AAT group (*p* < 0.01). ACC and FAS activities were higher in the VBT group than in the VAT group, while CPT-1α was lower (*p* < 0.01). On day 10 (Figure S3E), ACC and CPT-1α activities were significantly higher in the TS group than in the Con group (*p* < 0.05). Compared to the TS group, ACC activity in the ABT and VAT groups and CPT-1α in the VBT and AAT groups decreased, and CPT-1α increased in the ABT and VAT groups (*p* < 0.01). CPT-1α activity was higher in the ABT group than in the VBT group (*p* < 0.01), while ACC was higher in the AAT group than in the VAT group, with FAS and CPT-1α being lower (*p* < 0.01). Additionally, CPT-1α was significantly higher in the ABT group than in the AAT group (*p* < 0.01), and ACC was higher in the VBT group than in the VAT group, while CPT-1α was lower (*p* < 0.01).

### 2.4. Effects of AMC on the Expression of AMPKα-SREBP-1c/PPARα-Regulating Hepatic Lipid Metabolism in Transported Rats

**Figure 3:**
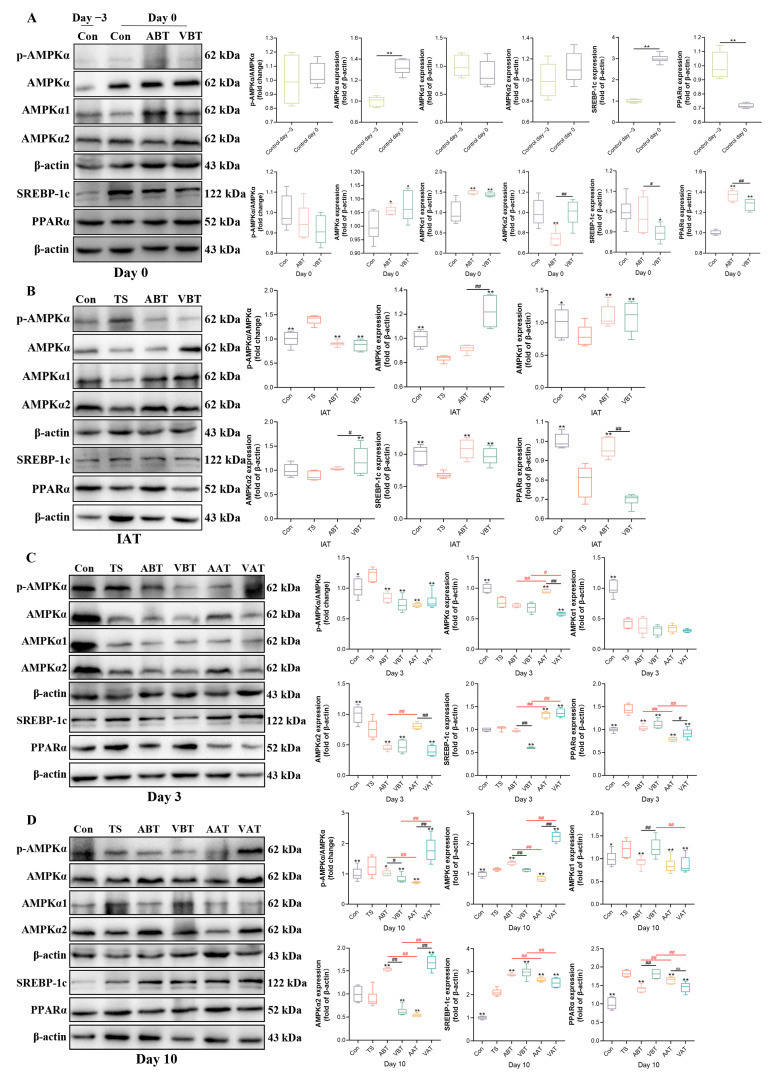
Effects of AMC on the protein levels of AMPKα-SREBP-1c/PPARα-regulating hepatic lipid metabolism in transported rats. (**A**–**D**) The protein levels of phosphorylated AMP-activated protein kinase alpha (p-AMPKα)/AMPKα, AMP-activated protein kinase alpha (AMPKα), AMPKα1, AMPKα2, sterol regulatory element-binding protein-1c (SREBP-1c), and Peroxisome proliferator-activated receptor alpha (PPARα) in the liver (**A**) on days −3 and 0, (**B**) at IAT, (**C**) on day 3, and (**D**) on day 10. Rats were assigned to one of six groups: Con, TS, ABT, VBT, AAT, and VAT groups. Data are expressed as the mean ± standard error of the mean (*n* = 6). * mean Con group on day −3 vs. day 0; Con vs. ABT or VBT on day 0; TS vs. Con, ABT, or VBT at IAT; and TS vs. Con, ABT, VBT, AAT, or VAT on days 3 and 10; # (black) mean ABT vs. VBT on days 0, 3, and 10 and at IAT, and AAT vs. VAT on days 3 and 10; # (red) mean ABT vs. AAT and VBT vs. VAT on days 3 and 10. *, # (black), and # (red): *p* < 0.05; **, ## (black), and ## (red): *p* < 0.01.

To investigate how AMC regulates hepatic lipid metabolism disorders in transported rats, we examined key genes and proteins related to lipid synthesis and fatty acid oxidation. As shown in Figure 3A, on day 0 compared to day −3, AMPKα and SREBP-1c protein levels increased significantly in the Con group (*p* < 0.01), while PPARα decreased (*p* < 0.01), with no significant changes in AMPKα phosphorylation or AMPKα1/2 levels (*p* > 0.05). In the ABT and VBT groups, AMPKα, AMPKα1, and PPARα protein levels were higher than in the Con group (*p* < 0.05 or *p* < 0.01), while AMPKα2 was significantly lower in the ABT group (*p* < 0.01). AMPKα2 was also lower in the ABT group than in the VBT group, while SREBP-1c and PPARα were higher (*p* < 0.05 or *p* < 0.01). AMPKα phosphorylation, AMPKα, and AMPKα1 levels were similar between ABT and VBT (*p* > 0.05). On day 0, CPT-1α mRNA levels decreased significantly in the Con group (*p* < 0.05) (Figure S4A,F), while other gene levels showed no significant change (*p* > 0.05). In the ABT group, SREBP-1c and CPT-1α mRNA levels were significantly higher than in the Con group (*p* < 0.01), but no significant differences were seen between Con and VBT (*p* > 0.05). SREBP-1c, PPARα, and CPT-1α mRNA levels were higher in the ABT group than in the VBT group (*p* < 0.05 or *p* < 0.01) (Figure S4B,G).

As shown in Figure 3B, at IAT, AMPKα phosphorylation was significantly higher in the TS group compared to the Con group (*p* < 0.01), while AMPKα, AMPKα1, SREBP-1c, and PPARα protein levels were significantly lower (*p* < 0.01). Compared to the TS group, AMPKα phosphorylation decreased in the ABT and VBT groups (*p* < 0.01), while AMPKα1 and SREBP-1c levels in both groups, PPARα in ABT, and AMPKα and AMPKα2 in VBT were significantly increased (*p* < 0.01). AMPKα and AMPKα2 were lower in ABT than in VBT (*p* < 0.05), while PPARα was higher (*p* < 0.01). AMPKα phosphorylation, AMPKα1, and SREBP-1c levels were similar between ABT and VBT (*p* > 0.05). As shown in Figure S4C,H, AMPKα1 mRNA increased in the TS group compared to the Con group (*p* < 0.05), while other gene levels were similar (*p* > 0.05). Compared to the TS group, PPARα in ABT and AMPKα1, AMPKα2, PPARα, and CPT-1α in VBT were significantly higher (*p* < 0.05 or *p* < 0.01). AMPKα1 and AMPKα2 mRNA levels were lower in ABT than in VBT (*p* < 0.01), while other gene levels were similar between ABT and VBT (*p* > 0.05).

As shown in Figure 3C, on day 3, the TS group had increased AMPKα phosphorylation and PPARα levels (*p* < 0.05), while AMPKα, AMPKα1, and AMPKα2 were decreased (*p* < 0.01) compared to the Con group, with no significant change in SREBP-1c (*p* > 0.05). Compared to the TS group, AMPKα phosphorylation, PPARα, AMPKα (in VAT), AMPKα2 (in ABT, VBT, and VAT), and SREBP-1c (in VBT) protein levels were significantly reduced in the ABT, VBT, AAT, and VAT groups (*p* < 0.01). AMPKα and SREBP-1c increased in the AAT and VAT groups, while SREBP-1c was higher in ABT than in VBT (*p* < 0.01). AMPKα, AMPKα2, and SREBP-1c were lower in ABT than in AAT, but PPARα was higher (*p* < 0.01). In VBT, AMPKα and PPARα were higher, and SREBP-1c was lower than in VAT (*p* < 0.01). Figure S4D,I show higher SREBP-1c, ACC1, FAS, and SCD-1 mRNA in the TS group compared to Con (*p* < 0.01). Various gene levels, such as AMPKα1, SREBP-1c, and PPARα, were raised in the ABT, VBT, AAT, and VAT groups (*p* < 0.05). SREBP-1c and ACC1 were lower in ABT compared to VBT, and AMPKα2 was lower in AAT than in VAT (*p* < 0.01).

As shown in Figure 3D, on day 10, the TS group had significantly increased AMPKα phosphorylation and higher levels of AMPKα, AMPKα1, SREBP-1c, and PPARα compared to the Con group (*p* < 0.01), with no change in AMPKα2 (*p* > 0.05). Compared to the TS group, AMPKα phosphorylation decreased in the ABT, VBT, and AAT groups, and protein levels of AMPKα, AMPKα1, and PPARα decreased in various groups (*p* < 0.01). AMPKα phosphorylation and levels of AMPKα and AMPKα2 were higher in ABT than VBT (*p* < 0.05), while AMPKα1 and PPARα were lower (*p* < 0.01). In the AAT group, AMPKα phosphorylation and levels of AMPKα and AMPKα2 were lower, but PPARα was higher than in VAT (*p* < 0.01). AMPKα phosphorylation and levels of AMPKα, AMPKα2, and SREBP-1c were higher in ABT than in AAT (*p* < 0.01), while PPARα was lower (*p* < 0.01). Compared to VAT, VBT had lower AMPKα phosphorylation and levels of AMPKα and AMPKα2 but higher AMPKα1, SREBP-1c, and PPARα (*p* < 0.01). As seen in Figure S4E,J, AMPKα2 mRNA was significantly higher in TS than Con (*p* < 0.01), with no differences in other mRNA levels. AMPKα2 mRNA was higher in VBT and AAT than TS (*p* < 0.05) and lower in ABT than VBT and AAT (*p* < 0.05). Other gene levels showed no differences between VBT and VAT (*p* > 0.05).

The effect of transportation and AMC/VitC addition on protein levels is shown in Figure S5. In the Con group, AMPKα phosphorylation remained unchanged on day 0 and at IAT but increased on days 3 and 10. AMPKα and SREBP-1c protein levels increased on day 0, decreased at IAT, and fluctuated during recovery. AMPKα1 decreased on day 0, increased at IAT, and showed similar fluctuations. AMPKα2 gradually increased from day −3 to day 10. PPARα decreased on day 0, increased at IAT, and gradually decreased during recovery. In the ABT group, AMPKα phosphorylation remained unchanged on day 0 and at IAT but increased during recovery. AMPKα and AMPKα1 decreased at IAT and recovered during the recovery period. AMPKα2 and PPARα increased at IAT and recovered during the recovery period. SREBP-1c increased on day 0, decreased at IAT, and gradually increased during recovery. In the VBT group, AMPKα phosphorylation remained stable on day 0 and at IAT but increased during recovery. AMPKα and AMPKα1 increased initially and then decreased, while AMPKα2 and SREBP-1c followed a similar pattern. PPARα decreased on days 0, 3, and at IAT but increased on day 10. In the TS group, AMPKα phosphorylation and AMPKα2 levels increased at IAT, while AMPKα and SREBP-1c decreased at IAT and fluctuated during recovery. AMPKα1 showed no changes at IAT but fluctuated during recovery, while PPARα increased at IAT, followed by fluctuations. In the AAT group, AMPKα phosphorylation, AMPKα1, and PPARα decreased on day 3 and increased on day 10. AMPKα, AMPKα2, and SREBP-1c increased on day 3 and then decreased. PPARα increased gradually during recovery. In the VAT group, AMPKα phosphorylation, AMPKα, AMPKα1, AMPKα2, and PPARα decreased on day 3 and increased on day 10, while SREBP-1c increased on day 3 and decreased on day 10.

The effect of transportation and AMC/VitC addition on mRNA levels is shown in Figure S6. In the Con group, AMPKα1 mRNA remained stable on day 0, decreased at IAT, and gradually increased on days 3 and 10. AMPKα2 gradually decreased on day 0 and at IAT, increased on day 3, and slightly decreased on day 10. SREBP-1c decreased on day 0, increased at IAT, decreased on day 3, and rose again on day 10. ACC1 and FAS increased on day 0, decreased at IAT, and gradually increased on days 3 and 10, while SCD-1 followed a similar pattern. PPARα increased on day 0, decreased at IAT, increased on day 3, and then decreased on day 10, while CPT-1α decreased on day 0, increased at IAT, and decreased again on days 3 and 10. In the ABT group, AMPKα1 and CPT-1α mRNA levels increased on day 0, decreased at IAT, and slightly increased on day 10 after a dip on day 3. AMPKα2 decreased on day 0, increased at IAT, and gradually declined on days 3 and 10. SREBP-1c, FAS, and PPARα increased on day 0, decreased at IAT, and declined again on day 10 after rising on day 3. ACC1 was stable on day 0, slightly decreased at IAT, and dropped on day 10 after rising on day 3, while SCD-1 gradually increased on days 0 and 3 and at IAT before declining on day 10. In the VBT group, AMPKα1, AMPKα2, and CPT-1α decreased on day 0, increased at IAT, and decreased on day 3 with a slight rise on day 10. SREBP-1c showed minor fluctuations, peaking on day 3. ACC1, PPARα, and FAS followed similar patterns, with fluctuations around day 3. SCD-1 increased on days 0 and 3 and at IAT before decreasing on day 10. In the TS group, AMPKα1 gradually decreased at IAT and on days 3 and 10, while AMPKα2 increased at IAT, rose on day 3, and decreased on day 10. SREBP-1c and ACC1 gradually increased at IAT and on day 3 but decreased on day 10. FAS and SCD-1 decreased at IAT, increased on day 3, and decreased again on day 10, while PPARα decreased at IAT and gradually increased on days 3 and 10. CPT-1α increased at IAT, decreased on day 3, and increased again on day 10. In the AAT group, AMPKα2, SREBP-1c, ACC1, FAS, SCD-1, and PPARα increased on day 3 and decreased on day 10, while AMPKα1 and CPT-1α gradually increased on days 3 and 10. In the VAT group, AMPKα1, AMPKα2, SREBP-1c, ACC1, FAS, SCD-1, PPARα, and CPT-1α all increased on day 3 and decreased on day 10.

## 3. Discussion

Transportation is an inevitable stressor in livestock farming, making animals more susceptible to diseases and resulting in economic losses [2]. Transportation stress can cause liver damage and lead to granular degeneration and steatosis in hepatocytes, as observed in pigs [36]. Serum TG, CHO, HDL, and LDL concentrations in beef cattle are decreased after short-distance transportation [9]. Additionally, transportation increased serum NEFA concentrations, peaking on the first day after transportation, suggesting enhanced fatty acid mobilization due to energy deficiency from feed restriction and increased energy need during transportation [10]. In this study, transportation was found to significantly alter serum lipid profiles, including reduced serum TG, VLDL-C, and NEFA concentrations and fatty droplets in the liver in the TS group at IAT. This may be related to the fact that the transportation process requires a large amount of energy from the body. Transportation leads to increased lipid mobilization, which provides the body with the necessary energy. Serum TC concentration was significantly lower in the TS group than in the Con group on day 3, but TG, TC, and LDL-C concentrations were significantly higher on day 10. Concurrently, fatty droplets in the liver of rats in the TS group were increased on days 3 and 10. These results suggest that transportation induces a transient disturbance in lipid metabolism, leading to elevated blood lipid levels and hepatic lipid accumulation, with partial recovery observed on the third day post transportation. The use of AMC in livestock has shown potential benefits, including mitigating heat-induced stress and enhancing growth performance in cattle [37]. Previous studies have demonstrated that AMC supplementation increases serum LDL-C and VLDL-C levels while reducing HDL-C levels in calves [16]. This study further explored the potential of AMC in alleviating transportation-induced lipid metabolism disorders. Rats supplemented with AMC showed improved blood lipid profiles and reduced hepatic fatty droplet accumulation compared to non-supplemented rats, indicating that AMC effectively mitigates transportation-induced hepatic lipid metabolism disturbances. Moreover, AMC was found to be more effective in reducing hepatic steatosis compared to vitamin C supplementation.

Key enzymes involved in lipid metabolism, such as FAS, ACC, and CPT-1α, play critical roles in regulating lipid synthesis and fatty acid oxidation in the liver [38,39,40]. The results indicated that transportation decreased hepatic ACC and FAS activities while increasing CPT-1α activity, suggesting a reduction in lipid anabolism and an increase in fatty acid oxidative metabolism. Compared with the Con group, hepatic ACC and CPT-1α activities were increased in the TS group on days 3 and 10. These indicate that both lipid synthesis and catabolism were elevated during the recovery period, reflecting a compensatory response to transportation-induced metabolic stress. Our preliminary study found that the addition of AMC reduced serum FAS and ACC enzyme activities in calves after transportation [16]. In this study, AMC supplementation reversed the alterations in hepatic-lipid-metabolizing enzymes, promoting lipid synthesis and modulating fatty acid oxidation, thereby restoring metabolic balance. Notably, AMC and VitC had opposite effects on CPT-1α activity. Compared with the VBT group, CPT-1α activity in the ABT group was higher. Compared with the VAT group, CPT-1α activity was lower in the AAT group. This suggests that AMC may regulate fatty acid oxidative metabolism more rapidly.

Stress-induced lipid metabolism disturbances are often associated with the AMPKα-SREBP-1c/PPARα pathway [15,41,42]. AMP-activated protein kinase (AMPK), an important regulator, maintains intracellular homeostasis by regulating lipid, glucose, and protein metabolism, autophagy, and mitochondrial biogenesis [43,44,45,46]. In this study, transportation increased AMPKα1 mRNA levels and AMPKα phosphorylation, while decreasing the relative protein levels of hepatic AMPKα, AMPKα1, SREBP-1c, and PPARα. This suggests that transportation promotes AMPKα activation, inhibits lipid synthesis, promotes hepatic fatty acid oxidation, and utilizes fat for energy immediately after transportation. AMC supplementation modulated these effects by inhibiting AMPKα activation, enhancing lipid synthesis, and reducing the oxidative catabolism of fatty acids, thus restoring lipid homeostasis. Compared with the TS group, AMPKα phosphorylation in the ABT group was significantly reduced, and the PPARα mRNA level and the relative protein levels of AMPKα, AMPKα1, SREBP-1c, and PPARα were elevated. These findings elucidate the role of AMC in regulating transportation-induced lipid metabolism disorders via the AMPKα-SREBP-1c/PPARα pathway. Further examination of different time points during the recovery period revealed that SREBP-1c, ACC, FAS, and SCD-1 mRNA levels, AMPKα phosphorylation, and the relative protein level of PPARα were elevated in the TS group on day 3. On day 10, AMPK phosphorylation and the relative protein levels of AMPKα, AMPKα1, SREBP-1c, and PPARα were increased, and the AMPKα2 mRNA level was decreased in the TS group. These indicate that both hepatic lipid synthesis and catabolism were enhanced in transported rats, with the recovery of hepatic lipid anabolism beginning by the third day post transportation. On days 3 and 10, hepatic AMPK phosphorylation and the relative protein level of PPARα were decreased in AMC-treated rats. On day 3, the relative protein level of SREBP-1c was decreased in the ABT group and increased in the AAT group, compared with the TS group. On day 10, the relative protein level of SREBP-1c was increased in the ABT and AAT groups, compared with the TS group. These suggest that AMC effectively maintained lipid synthesis and reduced fatty acid oxidation during this period by modulating the AMPKα pathway. These results highlight the potential of AMC as a therapeutic intervention to alleviate transportation stress and improve metabolic health in livestock.

## 4. Materials and Methods

### 4.1. Animals and Treatments

**Figure 4:**
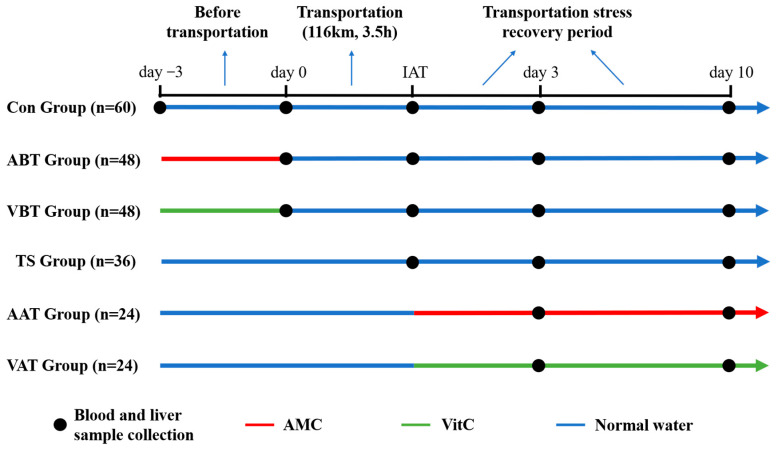
The design of experiment. AMC = alkaline mineral complex water, VitC = vitamin C. Rats were assigned to one of six groups: control group (Con), transportation stress group (TS), AMC addition before transportation group (ABT), VitC addition before transportation group (VBT), AMC addition after transportation group (AAT), and VitC addition after transportation group (VAT). IAT = immediately after transportation.

All procedures related to animals were conducted in accordance with the guidelines of the Animal Care and the Ethics Committee of Sichuan Agricultural University (Approval No. SYXK 2019–187). Male Sprague Dawley rats (SPF, 25 days old, 80 ± 10 g) were bought from Beijing SiPeiFu Biotechnology Co., Ltd. (Beijing, China). For 7 days preceding the experiment, the rats were housed at 26 ± 1 °C and 70% room humidity, with ad libitum access to food and water. Rats were randomly divided into 6 groups: control group (Con, 60 rats), transportation stress group (TS, 36 rats), AMC addition before transportation group (ABT, 48 rats), vitamin C (VitC) addition before transportation group (VBT, 48 rats), AMC addition after transportation group (AAT, 24 rats) and VitC addition after transportation group (VAT, 24 rats). Except for the Con group, all other groups underwent a transportation stress test, which lasted a total duration of 3.5 h, covered a distance of 116 km, and had an average speed of 42 km/h. During transportation, temperature increased by approximately 4 °C (from 15 to 19 °C) and humidity decreased by approximately 13% (from 62% to 49%). The Con and TS groups were fed normally both before and after transportation. ABT and VBT groups were administered with AMC or VitC in water for 3 days before transportation and then fed normally for 10 days after transportation. AAT and VAT groups were fed normally for 3 days before transportation and were given AMC or VitC for 10 days after transportation. Each cage housed four rats, and based on observations during the acclimation period, the daily water intake per cage exceeded 120 mL. AMC (0.75 mL/kg/day) or VitC (100 mg/kg/day) was dissolved in 120 mL of water, allowing the rats free access to drinking water. Based on changes in body weight and water intake, the actual average dose of AMC consumed was 0.77 mL/kg/day in the ABT group and 1.14 mL/kg/day in the AAT group. For VitC, the actual average dose consumed was 108.9 mg/kg/day in the VBT group and 139 mg/kg/day in the VAT group. The Con group was a baseline control, where rats were not exposed to transportation stress. This group provides standard physiological and health indicators under normal conditions, allowing a comparison to isolate the effects of transportation and treatments. The TS group evaluated transportation stress alone, with no supplementation, showing how stress impacts physiological and biochemical parameters without intervention. Comparing the TS group with the Con group and treatment groups (ABT, VBT, AAT, VAT) enables the assessment of transportation stress and the effectiveness of supplementation before or after stress exposure. Twelve rats were randomly selected for dissection on days −3, 0, 3, and 10 and immediately after transportation (IAT) (Figure 4). The rats were anesthetized with an intramuscular injection of Sutera 50 (brand name for an anesthetic). Blood was collected from the abdominal aorta, and liver tissues were quickly frozen with liquid nitrogen and stored at −80 °C. After allowing the whole blood to sit at room temperature for 2 h, it was centrifuged at 3500 rpm for 10 min at 4 °C to obtain serum. The serum was then stored at −80 °C.

### 4.2. Oil-Red O Staining

The liver tissue was collected and then dehydrated in a 15% sucrose solution at 4 °C. Subsequently, it was transferred to a 30% sucrose solution at 4 °C for further dehydration. The dehydrated tissues were OCT-embedded, sectioned at a thickness of 8–10 μm, and stored at −20 °C. The frozen sections were warmed and dried for 10 min, followed by fixation in 4% paraformaldehyde for 15 min. After fixation, the sections were rinsed three times with PBS for 5 min each time. The washed sections were immersed in the oil-red O working solution for 10–15 min, rinsed three times with PBS for 5 min each time, and then stained in the oil-red O working solution for 1–2 h at 37 °C. They were differentiated with 75% alcohol for 2 s and washed with water for 1 min. Finally, the surrounding water was absorbed with a paper towel and the slices were sealed with glycerol gelatin. The lipids in the liver were observed and photographed using a light microscope from Olympus Corporation in Tokyo, Japan.

### 4.3. Lipid Metabolism Characteristic Assay

Concentrations of serum TG, TC, HDL-C, LDL-C, and VLDL-C were detected through biochemical analysis using an autoanalyzer (BS2000M, Mindray, Shenzhen, China), with the original kits specifically designed for this equipment. The serum NEFA concentration and hepatic activities of ACC1, FAS, and carnitine palmitoyl transferase 1 (CPT-1α) were measured using commercial kits from Jiancheng Biotechnology Research Institute (Nanjing, China).

### 4.4. Quantitative Real-Time Polymerase Chain Reaction (qRT-PCR)

According to the manufacturer’s recommendations, total RNA was extracted from liver tissue powder stored at −80 °C using Trizol UP reagent (cat. no. ET111; TransGen Biotech, Beijing, China). cDNA was then synthesized using the AT341 Reverse Transcription Kit produced by TransGen Biotech according to the instructions. Then, qRT-PCR was performed in duplicate using the SYBR premix Ex Taq kit (cat. no. AQ601; TransGen Biotech, Beijing, China) and the Roche LightCycler^®^ 480 machine (Roche, Basel, Switzerland). The primer sequences are listed in Table 1. *β-actin* was used as a housekeeping gene for mRNA. Relative mRNA expression was calculated by the 2^−ΔΔCt^ method.

### 4.5. Western Blot Assays

Liver tissue was ground into powder with liquid nitrogen in a mortar; 40 mg of liver powder was added to 400 μL of RIPA lysis solution (containing 1% PMSF), sonicated and crushed at 12,000× *g*, and centrifuged at 4 °C for 5 min; and the supernatant was stored in portions at −80 °C. The protein concentration of the samples was determined by BCA. Equal amounts of protein were subjected to SDS-PAGE (10–12%). Protein was transferred to polyvinylidene difluoride membranes and immunoblotted with the primary antibodies. Antibodies are shown in Table 2. After washing with TBST, the membranes were incubated with a peroxidase-conjugated secondary antibody. Protein bands were detected with an ECL Plus kit (cat. no. PD203, Oriscience, Chengdu, China).

### 4.6. Statistical Analysis

The data were tested for normal distribution using the Shapiro–Wilk test and analyzed with two-way ANOVA and one-way ANOVA using SPSS 27.0 software and plotted using GraphPad Prism 8.0 software. Results were repeated in at least three independent experiments. All results are expressed as the mean ± standard error of the mean. *p* < 0.05 was considered significant, and *p* < 0.01 was considered highly significant.

## 5. Conclusions

**Figure 5:**
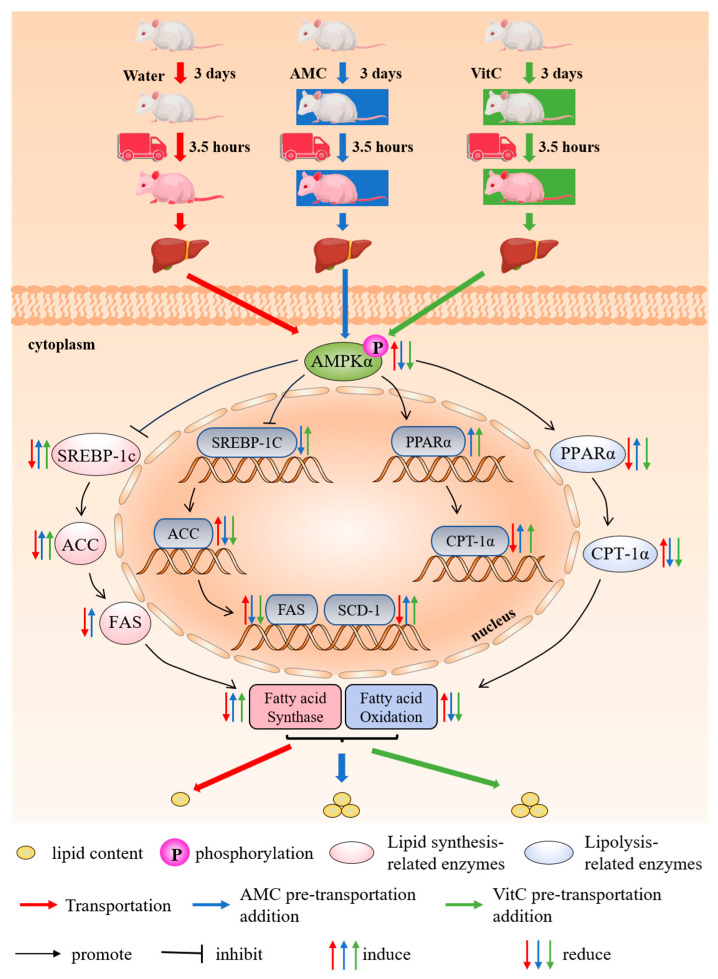
Alkaline mineral complex water (AMC) reduces transportation-induced hepatic lipid mobilization by AMPKα-SREBP-1c/PPARα pathway. Immediately after transportation, transportation decreases hepatic lipid anabolism and increases catabolism, leading to fatty acid depletion for energy. AMC and vitamin C inhibit AMPKα activation, balancing anabolism and catabolism and preventing fatty acid overuse.

**Figure 6:**
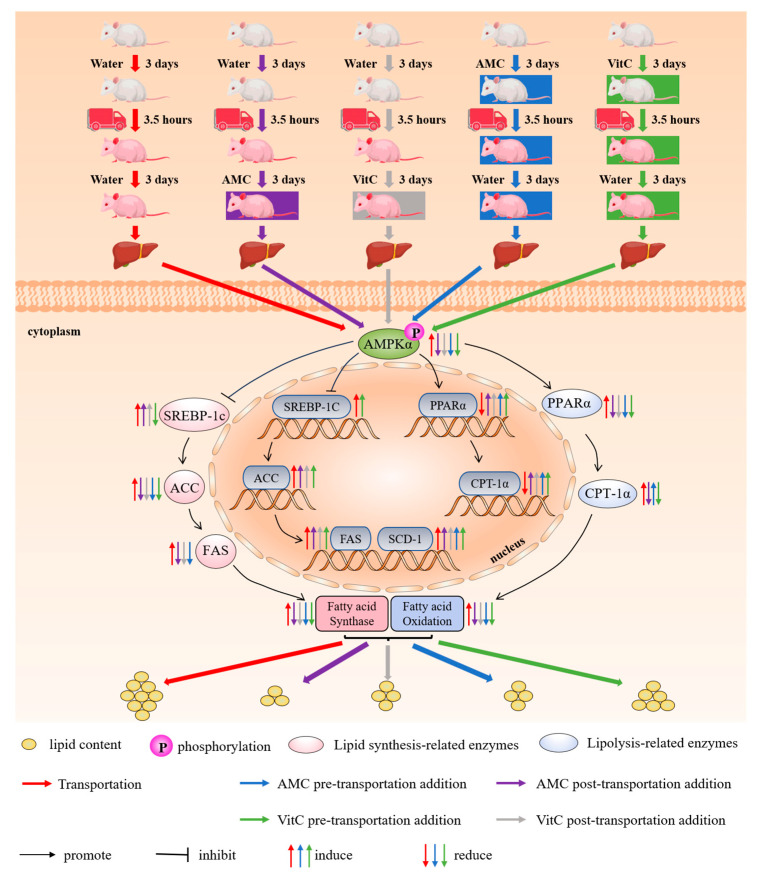
Alkaline mineral complex water (AMC) attenuates transportation-induced hepatic lipid metabolism dysregulation by AMPKα-SREBP-1c/PPARα pathway. On the third day after transportation, transportation disrupts lipid metabolism, causing deposition. Pre- and post-transportation addition of AMC reduced hepatic lipid anabolism and increased fatty acid catabolism. Vitamin C had the same regulatory effect. Notably, AMW supplementation was more effective.

This study demonstrates that transportation disrupts hepatic lipid metabolism in rats by activating the AMPKα-SREBP-1c/PPARα pathway, resulting in altered lipid profiles and enzyme activities that affect both lipid synthesis and catabolism. Specifically, transportation leads to a reduction in hepatic lipid anabolism and an increase in catabolism immediately after transportation, causing a depletion of fatty acids for energy (Figure 5). During the recovery period, these disruptions continue, characterized by increased lipid deposition (Figure 6). Supplementation with AMC was effective in mitigating these transportation-induced lipid metabolism disorders. AMC modulated the AMPKα-SREBP-1c/PPARα pathway, inhibiting the transportation-stress-induced activation of AMPKα and adjusting the expression of key genes and proteins such as SREBP-1c, ACC1, FAS, PPARα, and CPT-1α. By regulating these pathways, AMC was able to restore lipid homeostasis, reduce the oxidative catabolism of fatty acids, and maintain stable lipid levels during the recovery period. These findings suggest that AMC has significant potential to alleviate transportation stress in livestock by modulating hepatic lipid mobilization dysregulation and dyslipidemia, offering a promising strategy for improving animal welfare and reducing economic losses associated with transportation stress in animal farming.

## Figures and Tables

**Table 1:** Primers used for RT-qPCR.

Gene	Gene Bank ID	Primer Sequences (5′ → 3′)
*AMPKα1*	NM_019142.3	Forward: TTCGGGAAAGTGAAGGTGGG
		Reverse: GGTTCTGGATCTCTCTGCGG
*AMPKα2*	NM_023991.1	Forward: TCGGCAAAGTGAAGATTGGAGA
		Reverse: TCCAACAACATCTAAACTGCGA
*SREBP-1c*	NM_001276708.1	Forward: AGCTGATGGAGACAGGGAGT
		Reverse: GTGGTAGCCATGCTGGAACT
*ACC1*	NM_022193.2	Forward: GGCGGCTCTGGAGGTATATG
		Reverse: ATGTGGGCAGCATGAACTGA
*FAS*	NM_017332.2	Forward: CCCCTCACATCAAGTGGGAC
		Reverse: CTCGGAACTGGCGTCAATGT
*SCD-1*	NM_139192.2	Forward: ACACCTTGCTCTGGGGGATA
		Reverse: TCAGAGAACTTGTGGTGGGC
*PPARα*	NM_013196.2	Forward: TCGTGGAGTCCTGGAACTGA
		Reverse: CTTCAGTCTTGGCTCGCCTC
*CPT-1α*	NM_031559.2	Forward: CTGGGGAAGAGACAGACACC
		Reverse: CCATCGTGGTAGAGCCAGAC
*β-actin*	NM_031144.3	Forward: GGAGATTACTGCCCTGGCTCCTAGC
		Reverse: GGCCGGACTCATCGTACTCCTGCTT

**Table 2:** Antibodies used in this paper.

Antibody	Company	Cat Number	Dilution
AMPKα	Cell Signaling Technology	5831	1:1000
p-AMPKα	Cell Signaling Technology	2535	1:1000
AMPKα1	Cell Signaling Technology	5832	1:1000
AMPKα2	Abclonal	A14052	1:1000
PPARα	Boster	A00600-2	1:1000
SREBP-1c	Affinity	AF4728	1:1000
β-actin	TransGen Biotech	HC201	1:1000

## Data Availability

The data that support the findings of this study are available from the corresponding author upon reasonable request.

## Supplementary Materials

The following supporting information can be downloaded at https://www.mdpi.com/article/10.3390/ijms252111373/s1.
