# Supplementary material for: Alkaline Mineral Complex Water Attenuates Transportation-Induced Hepatic Lipid Metabolism Dysregulation by AMPKα-SREBP-1c/PPARα Pathways
Source: Int J Mol Sci. 2024 Oct 23;25(21):11373. doi: 10.3390/ijms252111373 (PMC11545688; doi:10.3390/ijms252111373)
Supplement: Supplementary file 1 [file ijms-25-11373-s001.zip › ijms-3242741-supplementary.pdf]

Supplementary material

1 Supplementary Figures

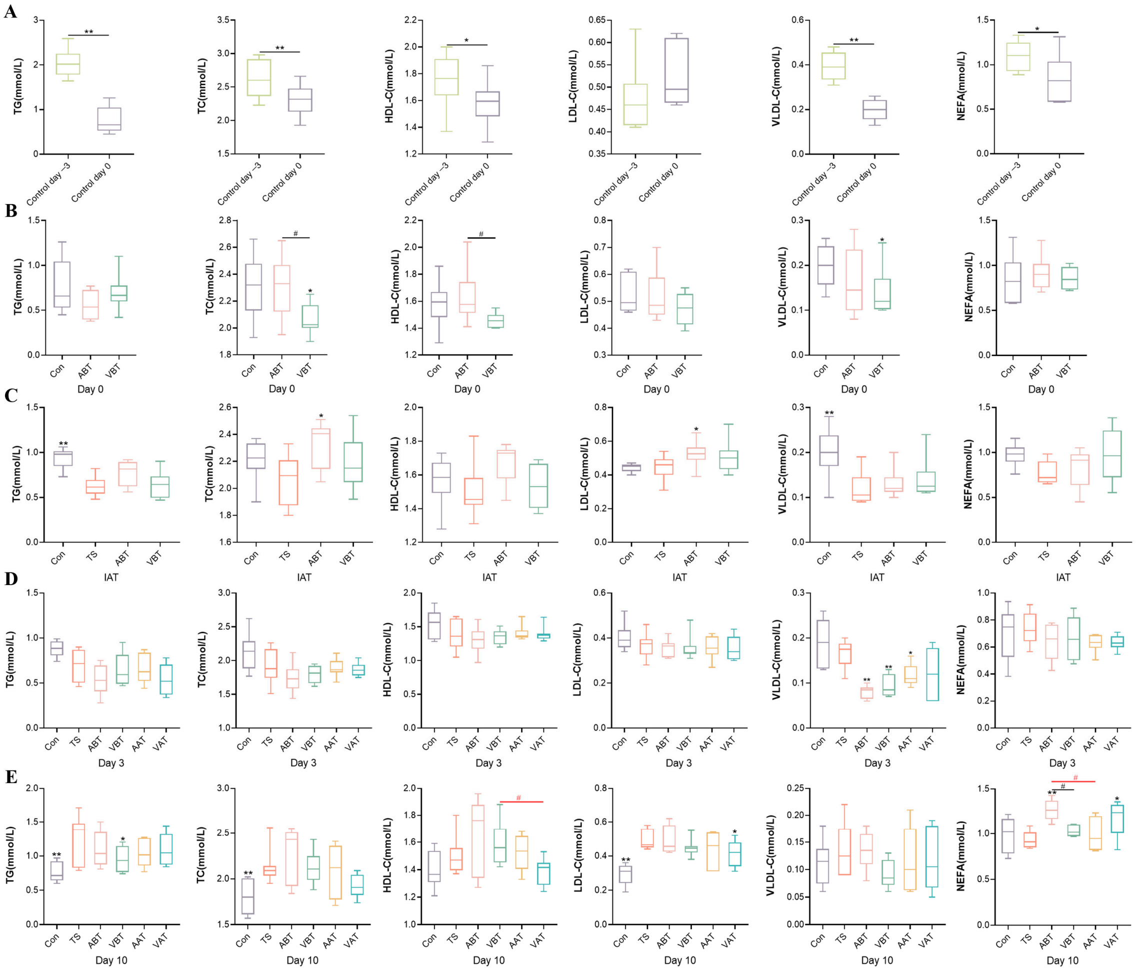

Figure S1. Effects of transportation and AMC and VitC addition on the concentrations of blood lipids. (A-E) The concentrations of TG, TC, HDL-C, LDL-C, VLDL-C, and NEFA (A) on day −3, (B) on day 0, (C) at IAT, (D) on day 3, and (E) on day 10. Rats were assigned to one of six groups: Con, TS, ABT, VBT, AAT, and VAT groups. Data are expressed as the mean ± standard error of the mean, where NEFA is n=6 and the rest are n =8. * mean Con group on day −3 vs. day 0; Con vs. ABT or VBT on day 0; TS vs. Con, ABT, or VBT at IAT; and TS vs. Con, ABT, VBT, AAT, or VAT on days 3 and 10; # (black) mean ABT vs. VBT on days 0, 3, and 10 and at IAT, and AAT vs. VAT on days 3 and 10; # (red) mean ABT vs. AAT and VBT vs. VAT on days 3 and 10. *, # (black), and # (red): $p < 0.05$; **, ## (black), and ## (red): $p < 0.01$.

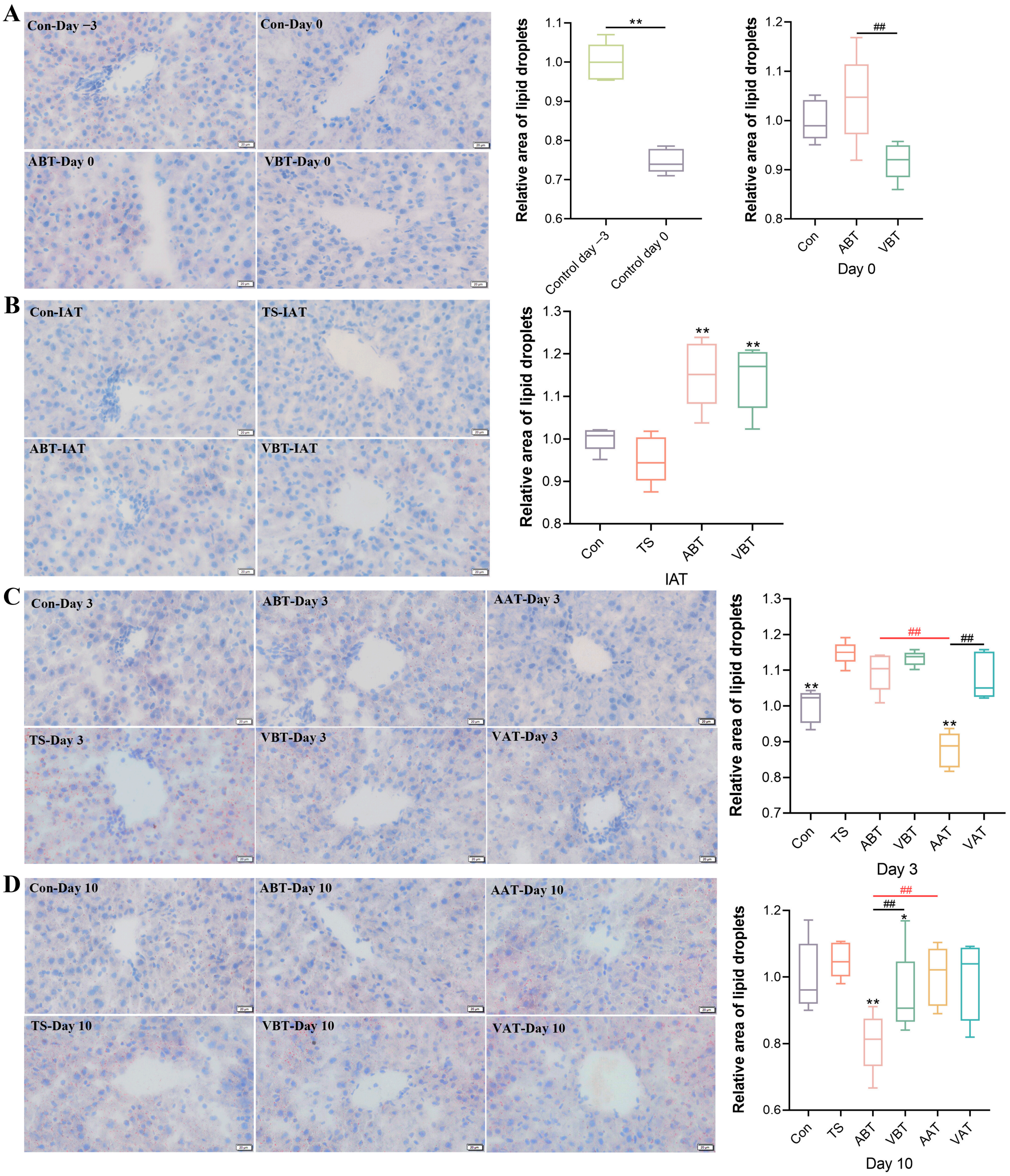

Figure S2. Effect of AMC on hepatic lipid accumulation in transported rats (×200). The picture shows the oil-red O staining of liver tissue sections (A) on day −3 and day 0, (B) at IAT, (C) on day 3, and (D) on day 10. Rats were assigned to one of six groups: Con, TS, ABT, VBT, AAT, and VAT groups. Data are expressed as the mean ± standard error of the mean.* mean Con group on day −3 vs. day 0; Con vs. ABT or VBT on day 0; TS vs. Con, ABT, or VBT at IAT; and TS vs. Con, ABT, VBT, AAT, or VAT on days 3 and 10; # (black) mean ABT vs. VBT on days 0, 3, and 10 and at IAT, and AAT vs. VAT on days 3 and 10; # (red) mean ABT vs. AAT and VBT vs. VAT on days 3 and 10. *, # (black), and # (red): $p < 0.05$; **, ## (black), and ## (red): $p < 0.01$.

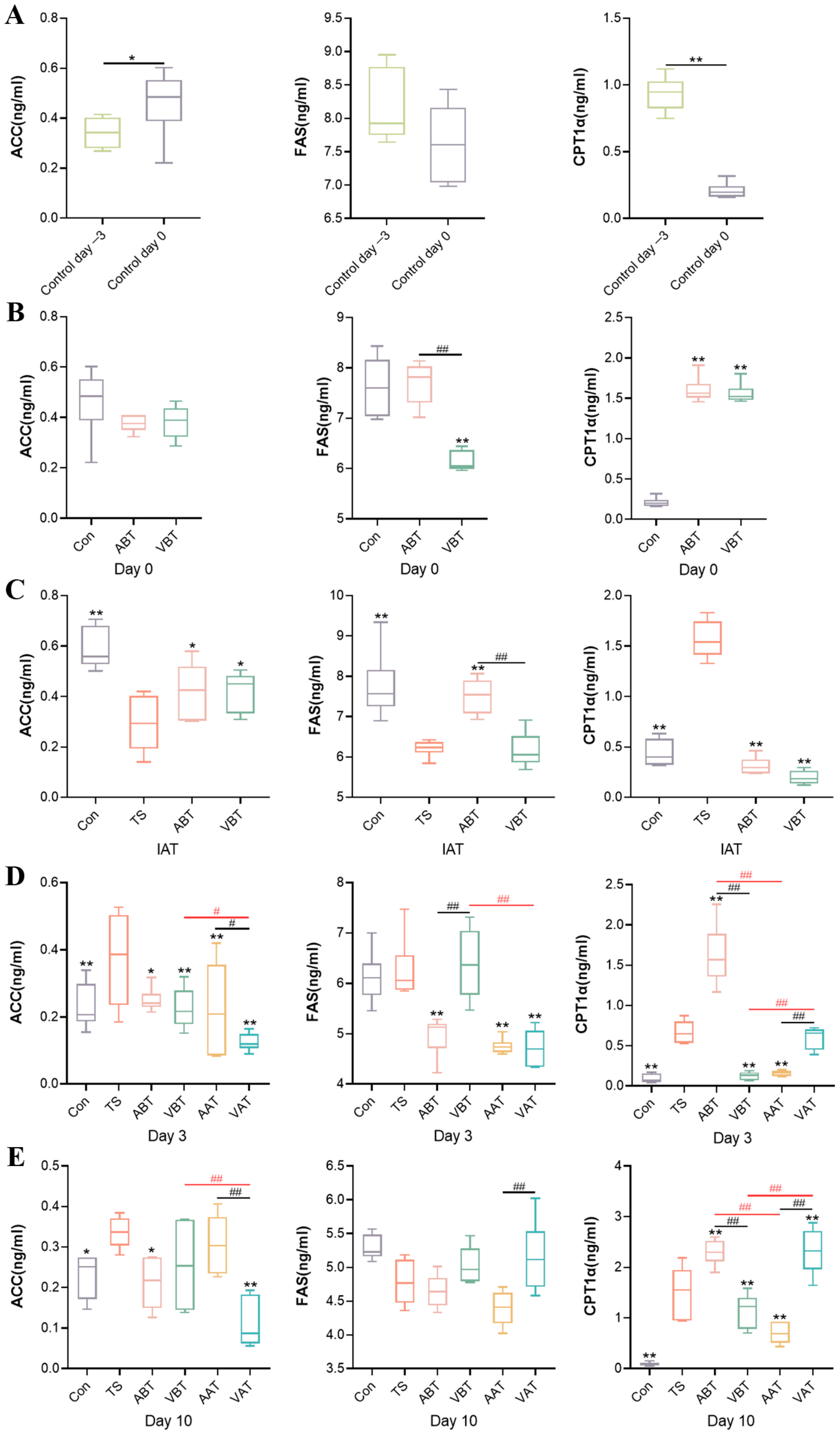

Figure S3. Effect of AMC on lipid-metabolizing enzymes in transported rats. (A-E) The activities of hepatic ACC, FAC, and CPT-1α (A) on day −3, (B) on day 0, (C) at IAT, (D) on day 3, and (E) on day 10. Rats were assigned to one of six groups: Con, TS, ABT, VBT, AAT, and VAT groups. Data are expressed as the mean ± standard error of the mean (n=6). * mean Con group on day −3 vs. day 0; Con vs. ABT or VBT on day 0; TS vs. Con, ABT, or VBT at IAT; and TS vs. Con, ABT, VBT, AAT, or VAT on days 3 and 10; # (black) mean ABT vs. VBT on days 0, 3, and 10 and at IAT, and AAT vs. VAT on days 3 and 10; # (red) mean ABT vs. AAT and VBT vs. VAT on days 3 and 10. *, # (black), and # (red): $p < 0.05$; **, ## (black), and ## (red): $p < 0.01$.

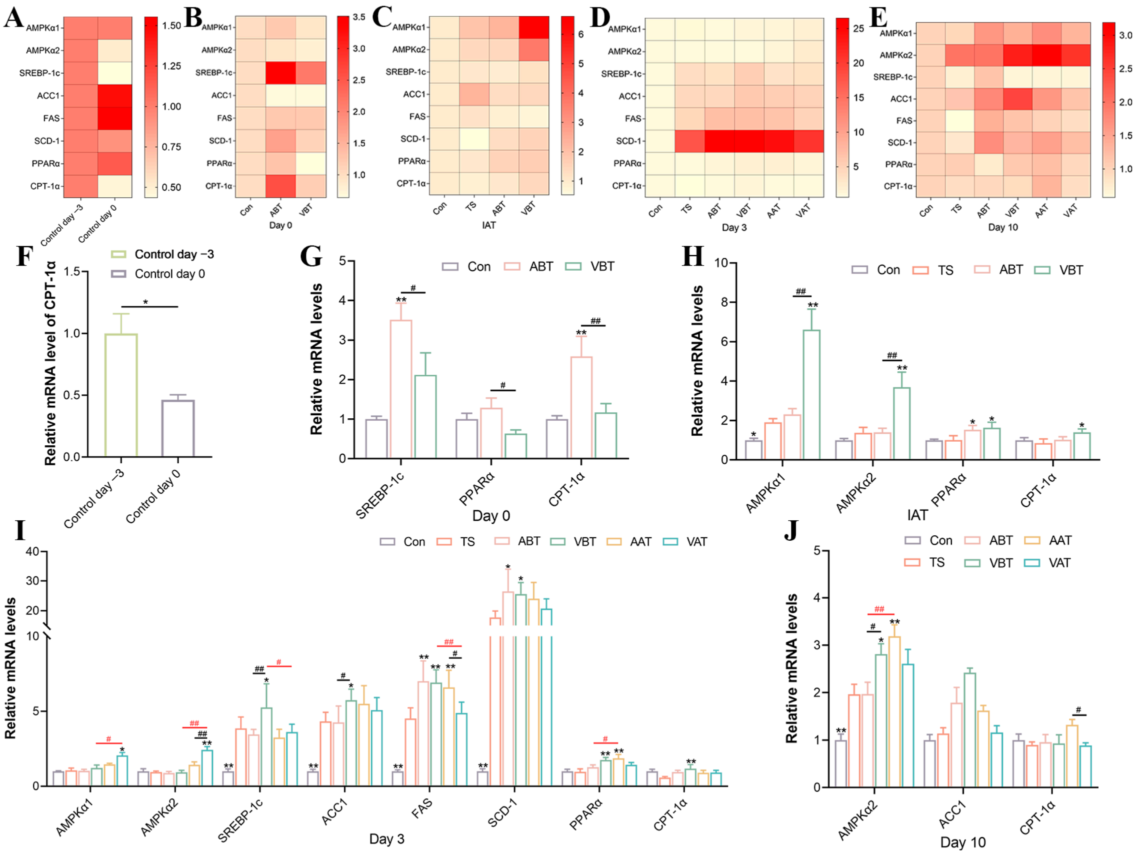

Figure S4. Effects of AMC on the mRNA expressions of AMPKα-SREBP-1c/PPARα pathway. (A-J) The mRNA expressions of AMPKα1, AMPKα2, SREBP-1c, ACC1, FAS, SCD-1, PPARα, and CPT-1α (A and F) on day −3, (B and G) on day 0, (C and H) at IAT, (D and I) on day 3, and (E and J) on day 10. Rats were assigned to one of six groups: Con, TS, ABT, VBT, AAT, and VAT groups. Data are expressed as the mean ± standard error of the mean (*n*=6). * mean Con group on day −3 vs. day 0; Con vs. ABT or VBT on day 0; TS vs. Con, ABT, or VBT at IAT; and TS vs. Con, ABT, VBT, AAT, or VAT on days 3 and 10; # (black) mean ABT vs. VBT on days 0, 3, and 10 and at IAT, and AAT vs. VAT on days 3 and 10; # (red) mean ABT vs. AAT and VBT vs. VAT on days 3 and 10. *, # (black), and # (red): $p < 0.05$; **, ## (black), and ## (red): $p < 0.01$.

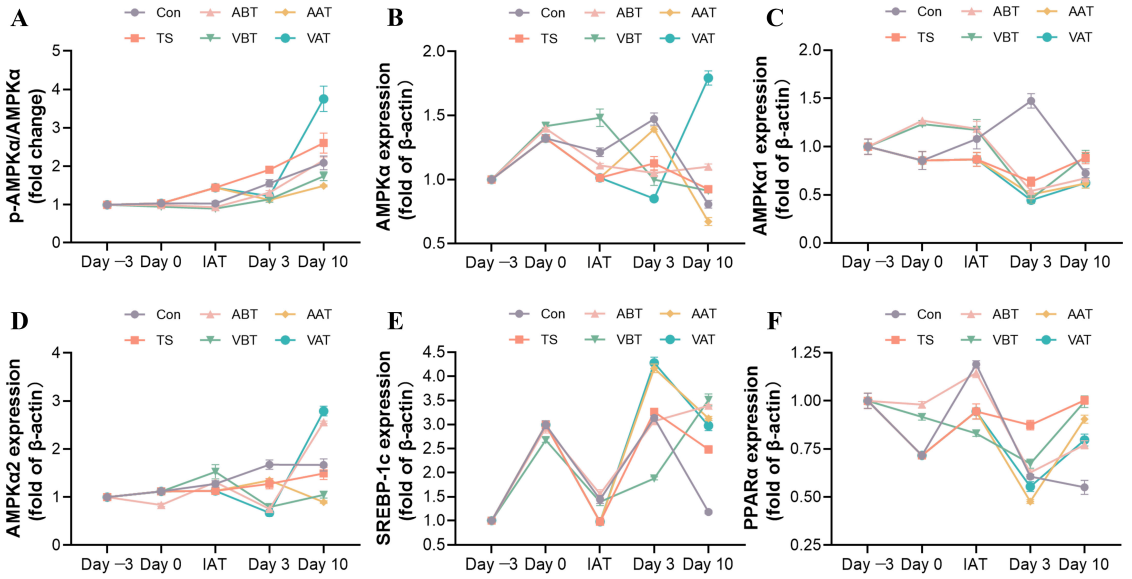

Figure S5. The effect of AMC and VitC on protein levels of AMPKα-SREBP-1c/PPARα pathway. (A) p-AMPKα, (B) AMPKα, (C) AMPKα1, (D) AMPKα2, (E) SREBP-1c, and (F) PPARα. Rats were assigned to one of six groups: Con, TS, ABT, VBT, AAT, and VAT groups. Data are expressed as the mean ± standard error of the mean (*n*=6).

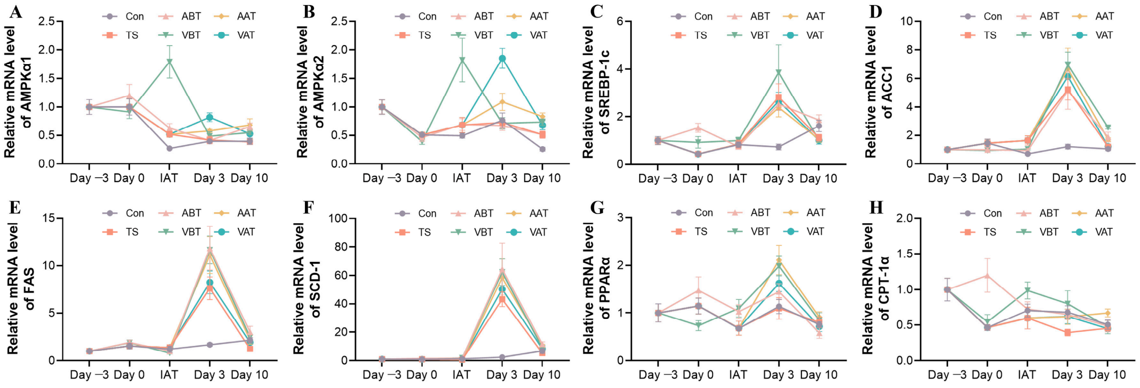

Figure S6. The effect of AMC and VitC on mRNA expressions of AMPKα-SREBP-1c/PPARα pathway. (A) AMPKα1, (B) AMPKα2, (C) SREBP-1c, (D) ACC1, (E) FAS, (F) SCD-1, (G) PPARα, and CPT-1α. Rats were assigned to one of six groups: Con, TS, ABT, VBT, AAT, and VAT groups. Data are expressed as the mean ± standard error of the mean (*n*=6).
